# Correction: Wu et al. WIP1 Inhibition by GSK2830371 Potentiates HDM201 Through Enhanced p53 Phosphorylation and Activation in Liver Adenocarcinoma Cells. *Cancers* 2021, *13*, 3876

**DOI:** 10.3390/cancers18030530

**Published:** 2026-02-06

**Authors:** Chiao-En Wu, Ahmed Khairallah Mahdi, Chen-Yang Huang, Chiao-Ping Chen, Yi-Ru Pan, John Wen-Cheng Chang, Jen-Shi Chen, Chun-Nan Yeh, John Lunec

**Affiliations:** 1 Division of Haematology-Oncology, Department of Internal Medicine, Chang Gung Memorial Hospital, Linkou Branch, Chang Gung University College of Medicine, Taoyuan 333, Taiwan; 8805017@cgmh.org.tw (C.-E.W.); b9202070@cgmh.org.tw (C.-Y.H.); D000017242@cgu.edu.tw (C.-P.C.); wen1902@cgmh.org.tw (J.W.-C.C.); js1101@adm.cgmh.org.tw (J.-S.C.); 2 Liver Research Center, Chang Gung Memorial Hospital, Linkou, Taoyuan 333, Taiwan; panyiru0331@cgmh.org.tw; 3 Department of Pathology and Forensic Medicine, College of Medicine, Al-Nahrain University, Baghdad 10006, Iraq; dr.ahmedkhairallah@nahrainuniv.edu.iq; 4 Newcastle University Cancer Centre, Bioscience Institute, Medical Faculty, Newcastle University, Newcastle upon Tyne NE2 4HH, UK; 5 Department of General Surgery, Chang Gung Memorial Hospital, Linkou Branch, Chang Gung University, Taoyuan 333, Taiwan

## Missing Funding

In the original publication [1], funding from the Iraqi Ministry of Higher Education and Scientific Research to Ahmed Khairallah Mahdi was not included. 

## Addition of an Author

Ahmed Khairallah Mahdi was not included as an author in the original publication. The corrected Author Contributions statement appears here.

**Author Contributions:** Conceptualization, C.-E.W., A.K.M., C.-Y.H., C.-N.Y. and J.L.; Data curation, C.-E.W. and C.-P.C.; Formal analysis, C.-P.C. and Y.-R.P.; Funding acquisition, C.-E.W. and C.-N.Y.; Investigation, C.-P.C., Y.-R.P., J.W.-C.C. and J.-S.C.; Methodology, C.-E.W., A.K.M., C.-Y.H. and Y.-R.P.; Project administration, C.-E.W.; Supervision, J.L.; Validation, C.-E.W., C.-Y.H., C.-N.Y. and J.L.; Visualization, C.-P.C.; Writing—Original draft, C.-E.W.; Writing—Review and editing, C.-N.Y. and J.L. All authors have read and agreed to the published version of the manuscript.

Ahmed Khairallah Mahdi and Chiao-En Wu have equally contributed to the initiation, conceptualization and methodology of the study.

The authors state that the scientific conclusions are unaffected. This correction was approved by the Academic Editor. The original publication has also been updated.
