# Supplementary material for: WIP1 Inhibition by GSK2830371 Potentiates HDM201 through Enhanced p53 Phosphorylation and Activation in Liver Adenocarcinoma Cells
Source: Cancers (Basel). 2021 Jul 31;13(15):3876. doi: 10.3390/cancers13153876 (PMC8345393; doi:10.3390/cancers13153876)
Supplement: Supplementary file 1 [file cancers-13-03876-s001.zip › Figure S10. Western Blot images.pdf]

Fig. 4A

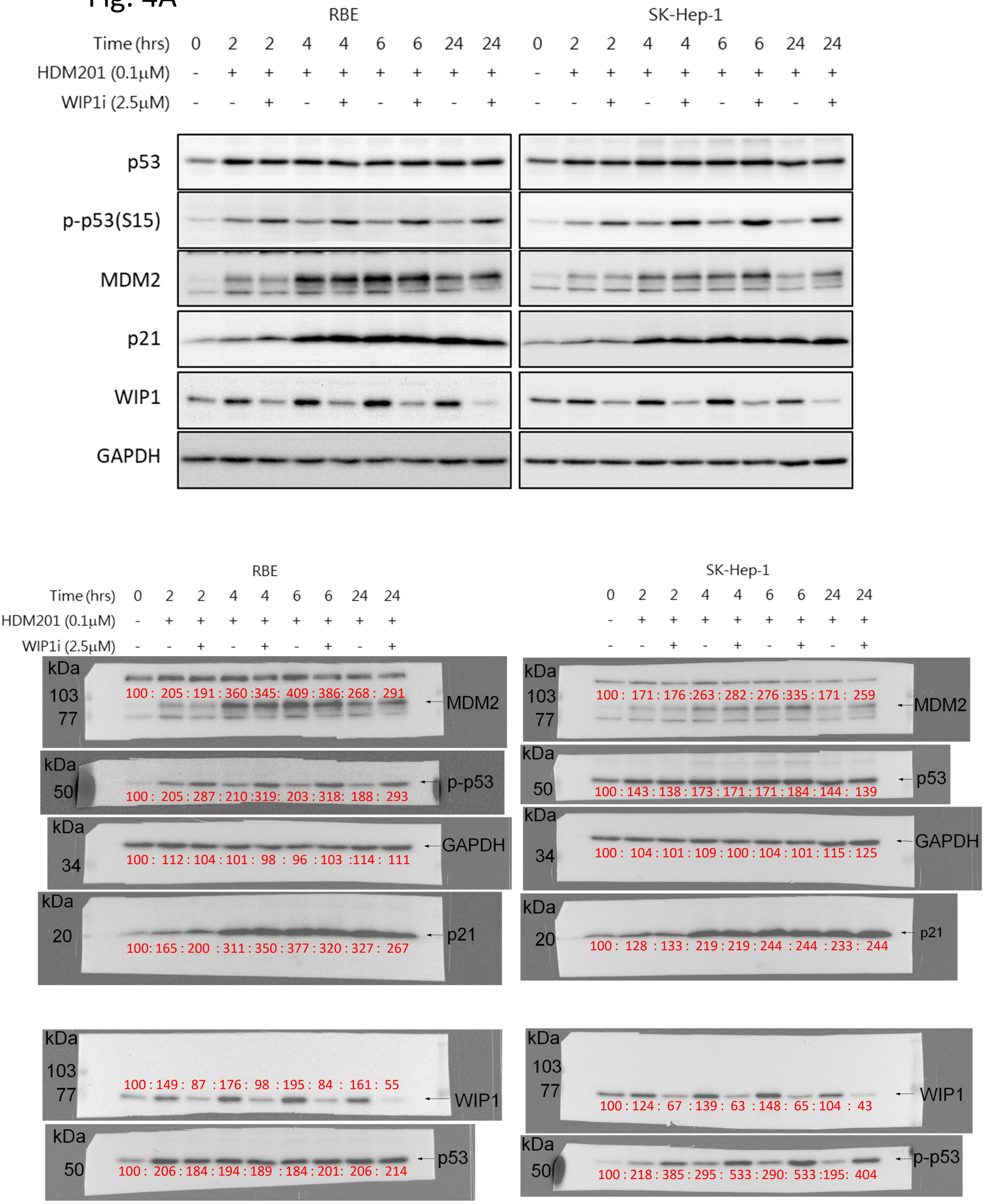
RBE
SK-Hep-1
Time (hrs) 0 2 2 4 4 6 6 24 24
HDM201 (0.1μM) - + + + + + + + +
WIP1i (2.5μM) - - + - + - + - +
p53
p-p53(S15)
MDM2
p21
WIP1
GAPDH
kDa
103
77
100 : 205 : 191 : 360 :345: 409 : 386: 268 : 291
MDM2
50
100 : 205 : 287 : 210 :319: 203 : 318: 188 : 293
p-p53
34
100 : 112 : 104 : 101 : 98 : 96 : 103 :114 : 111
GAPDH
20
100: 165 : 200 : 311 : 350 : 377 : 320 : 327 : 267
p21
100 : 149 : 87 : 176 : 98 : 195 : 84 : 161 : 55
WIP1
100 : 206 : 184 : 194 : 189 : 184 : 201: 206 : 214
p53
100 : 171 : 176 :263 : 282 : 276 :335 : 171 : 259
100 : 143 : 138 : 173 : 171 : 171 : 184 : 144 : 139
100 : 104 : 101 : 109 : 100 : 104 : 101 : 115 : 125
100 : 128 : 133 : 219 : 219 : 244 : 244 : 233 : 244
100 : 124 : 67 : 139 : 63 : 148 : 65 : 104 : 43
100 : 218 : 385 : 295 : 533 : 290: 533 :195: 404

Fig. 4B

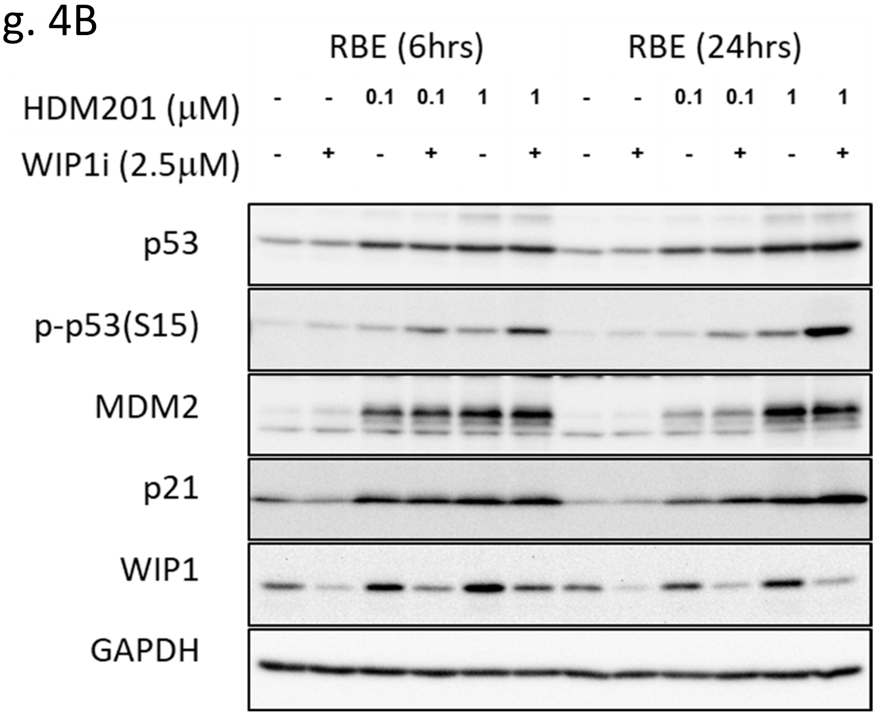
RBE (6hrs)
RBE (24hrs)
HDM201 (μM) - - 0.1 0.1 1 1 - - 0.1 0.1 1 1
WIP1i (2.5μM) - + - + - + - + - + - +
p53
p-p53(S15)
MDM2
p21
WIP1
GAPDH

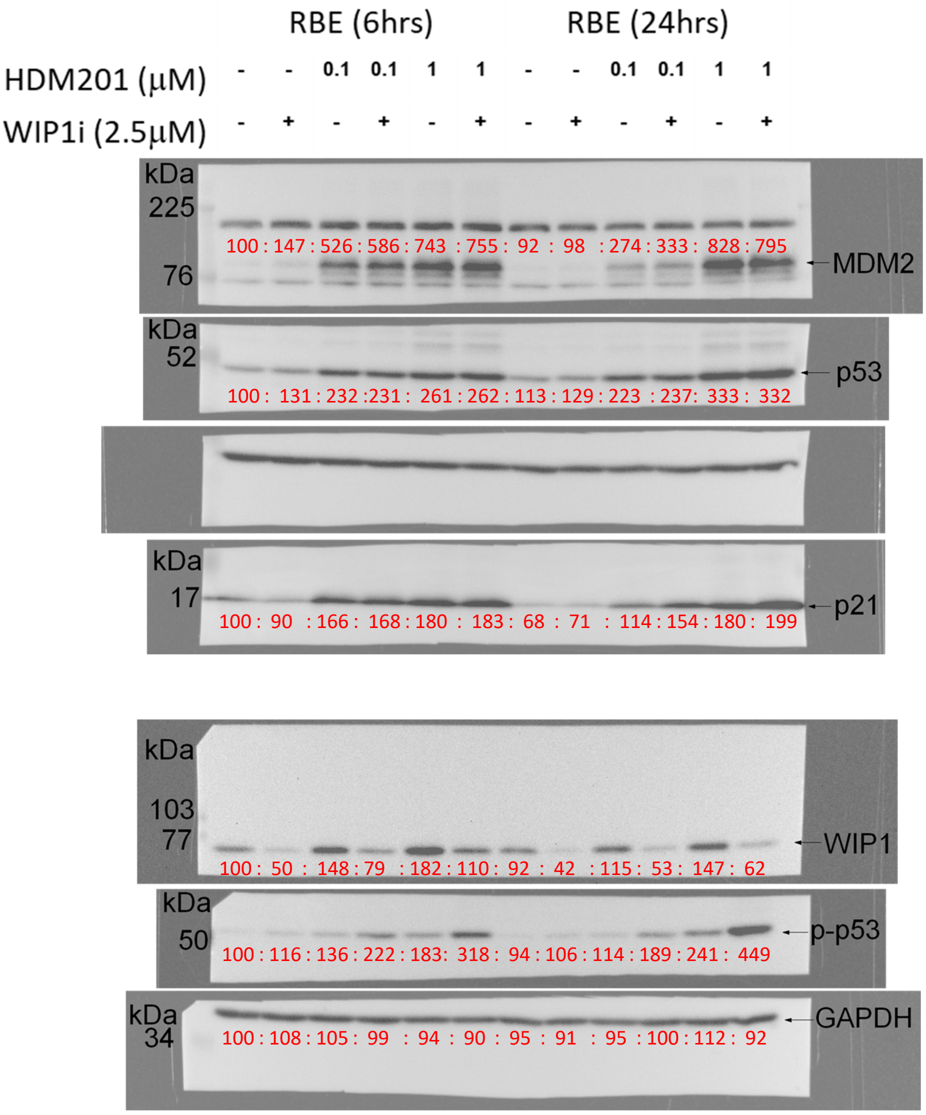
RBE (6hrs)
RBE (24hrs)
HDM201 (μM) - - 0.1 0.1 1 1 - - 0.1 0.1 1 1
WIP1i (2.5μM) - + - + - + - + - + - +
kDa
225
76
100 : 147 : 526 : 586 : 743 : 755 : 92 : 98 : 274 : 333 : 828 : 795
MDM2
kDa
52
100 : 131 : 232 :231 : 261 : 262 : 113 : 129 : 223 : 237: 333 : 332
p53
kDa
17
100 : 90 : 166 : 168 : 180 : 183 : 68 : 71 : 114 : 154 : 180 : 199
p21
kDa
103
77
100 : 50 : 148 : 79 : 182 : 110 : 92 : 42 : 115 : 53 : 147 : 62
WIP1
kDa
50
100 : 116 : 136 : 222 : 183: 318 : 94 : 106 : 114 : 189 : 241 : 449
p-p53
kDa
34
100 : 108 : 105 : 99 : 94 : 90 : 95 : 91 : 95 : 100 : 112 : 92
GAPDH

Fig. 4D

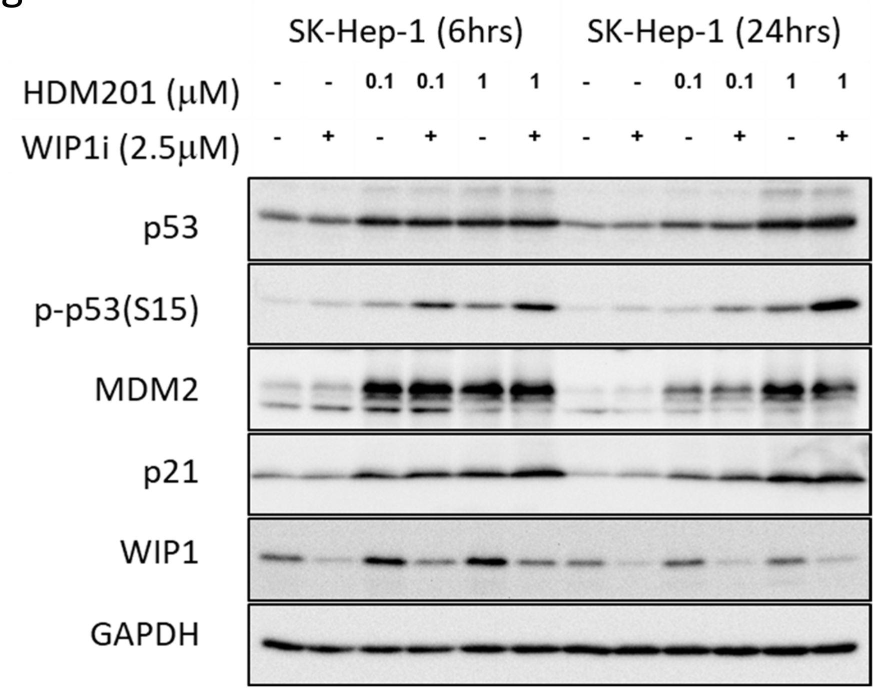

SK-Hep-1 (6hrs)
SK-Hep-1 (24hrs)
HDM201 (μM) - - 0.1 0.1 1 1 - - 0.1 0.1 1 1
WIP1i (2.5μM) - + - + - + - + - + - +
p53
p-p53(S15)
MDM2
p21
WIP1
GAPDH


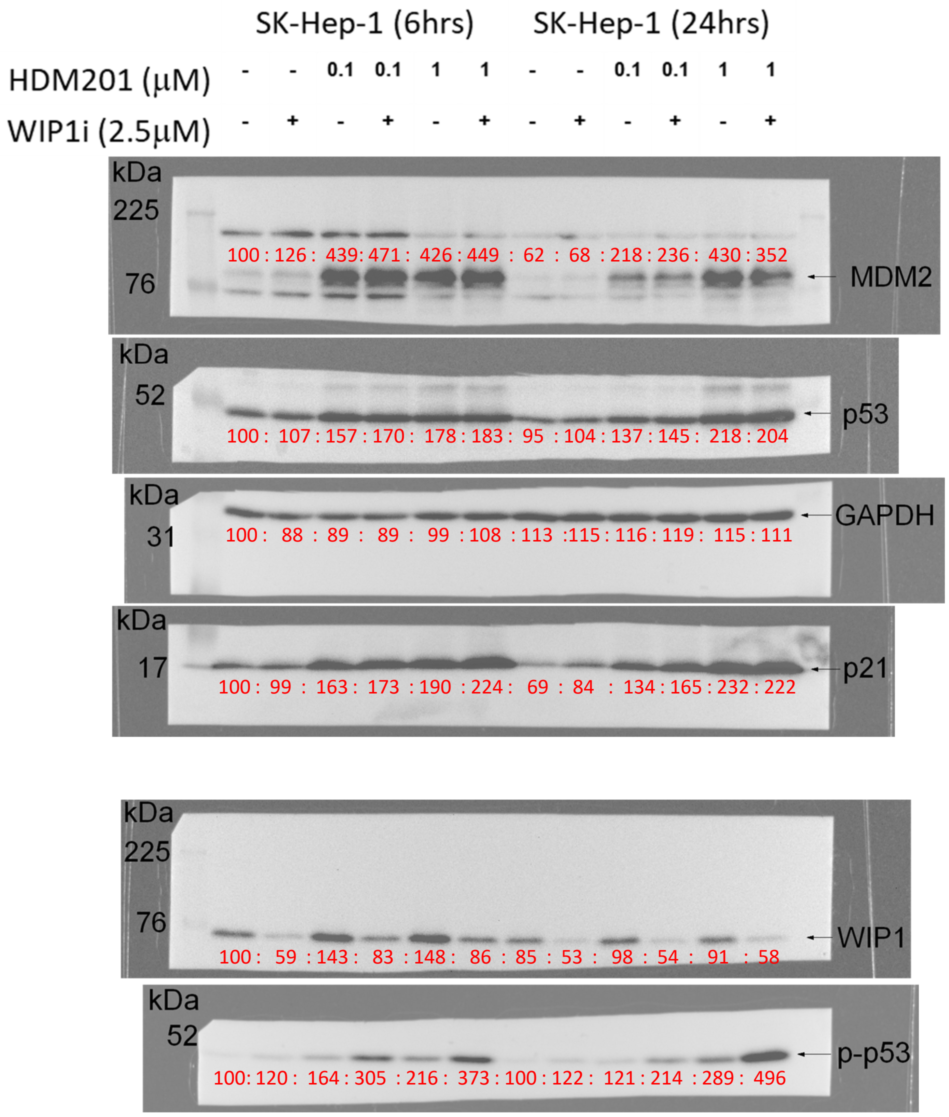

SK-Hep-1 (6hrs)
SK-Hep-1 (24hrs)
HDM201 (μM) - - 0.1 0.1 1 1 - - 0.1 0.1 1 1
WIP1i (2.5μM) - + - + - + - + - + - +
kDa
225
76
100 : 126 : 439: 471 : 426 : 449 : 62 : 68 : 218 : 236 : 430 : 352
MDM2
kDa
52
100 : 107 : 157 : 170 : 178 : 183 : 95 : 104 : 137 : 145 : 218 : 204
p53
kDa
31
100 : 88 : 89 : 89 : 99 : 108 : 113 :115 : 116 : 119 : 115 : 111
GAPDH
kDa
17
100 : 99 : 163 : 173 : 190 : 224 : 69 : 84 : 134 : 165 : 232 : 222
p21
kDa
225
76
100 : 59 : 143 : 83 : 148 : 86 : 85 : 53 : 98 : 54 : 91 : 58
WIP1
kDa
52
100: 120 : 164 : 305 : 216 : 373 : 100 : 122 : 121 : 214 : 289 : 496
p-p53
