# Supplementary material for: WIP1 Inhibition by GSK2830371 Potentiates HDM201 through Enhanced p53 Phosphorylation and Activation in Liver Adenocarcinoma Cells
Source: Cancers (Basel). 2021 Jul 31;13(15):3876. doi: 10.3390/cancers13153876 (PMC8345393; doi:10.3390/cancers13153876)
Supplement: Supplementary file 1 [file cancers-13-03876-s001.zip › Supplementary figures.pdf]

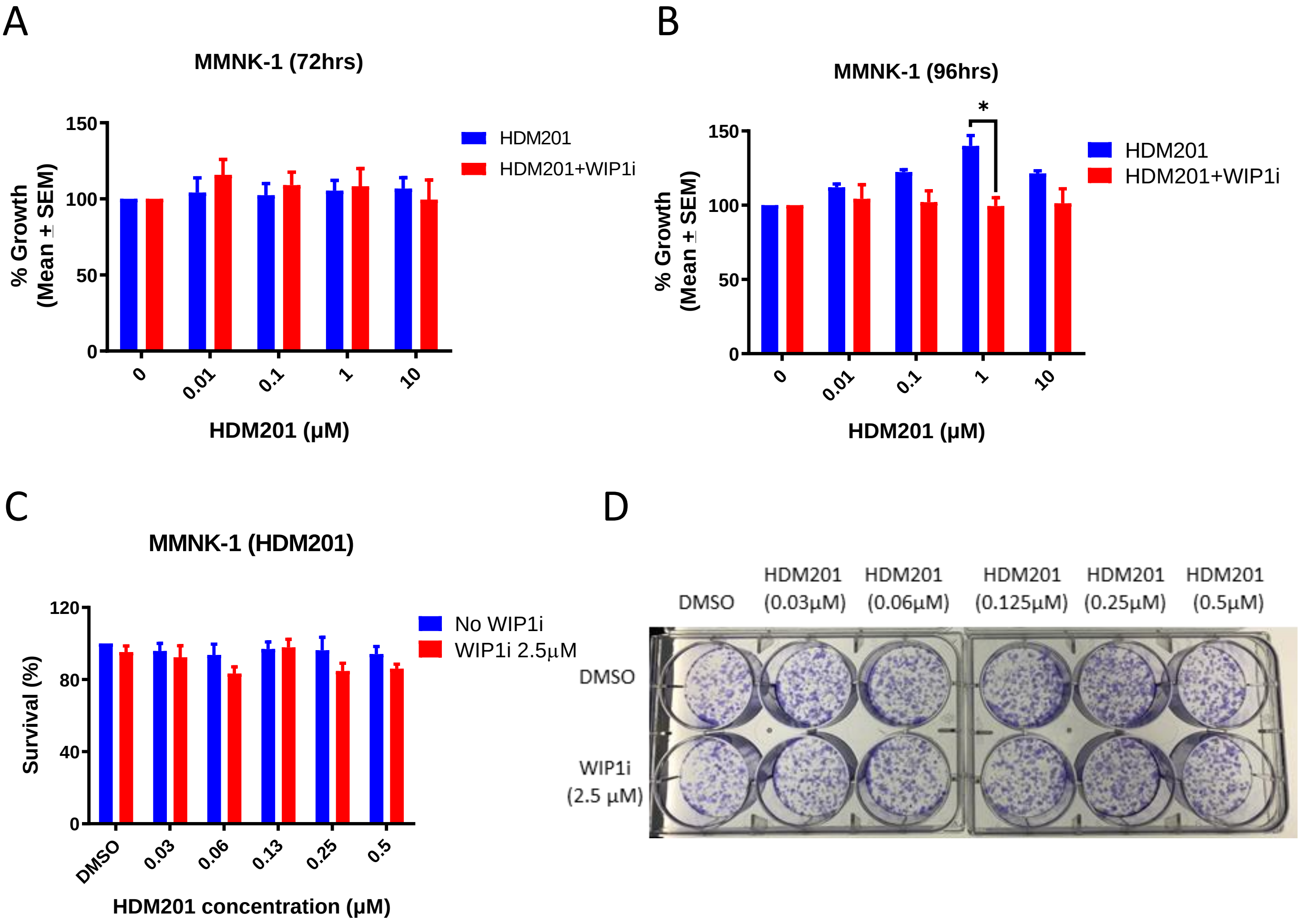

Supplementary Figure S1. GSK2830371 not enhanced cell and colony inhibition by HDM201 in MMNK-1. MMNK-1 as a normal liver cell was treated with 0-1 µM HDM201 alone or in combination with 2.5 µM WIP1 inhibitor (WIP1i) in 0.5% DMSO for 72 (A) and 96 (B) hours. (C, D) Clonogenic assay in MMNK-1 was tested with 0-0.5 µM HDM201 alone or in combination with WIP1i. The percentage of survival was normalized to DMSO. Panels A, B, and C showed the mean ± SEM from three independent duplicate experiments.. *, p<0.05.

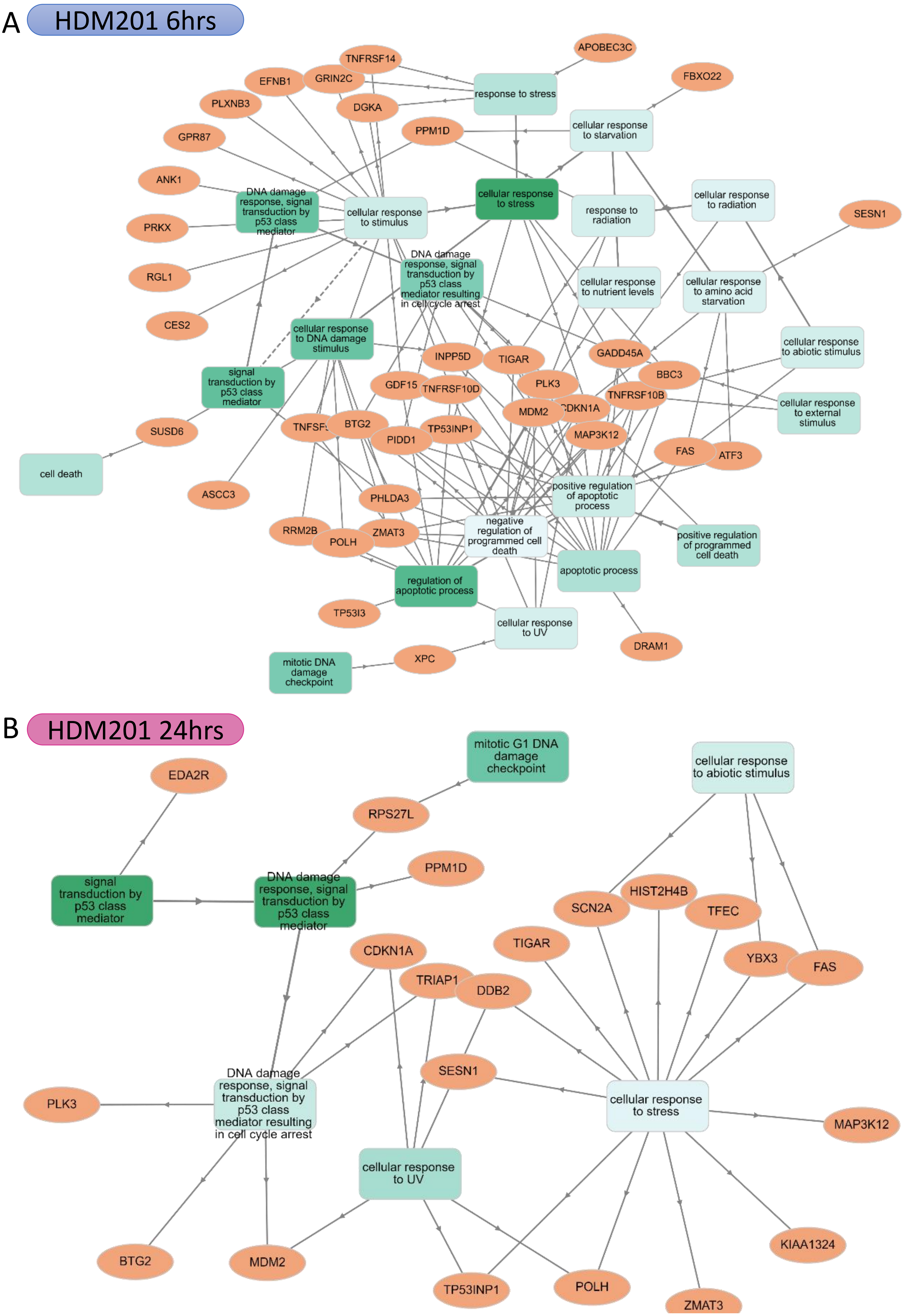

Supplementary Figure S2. RNAseq for Gene ontology (GO) enrichment analysis with Database of Immune Cell Expression (DICE). RBE were treated with HDM201 alone for 6 hours (A) and 24 hors (B).

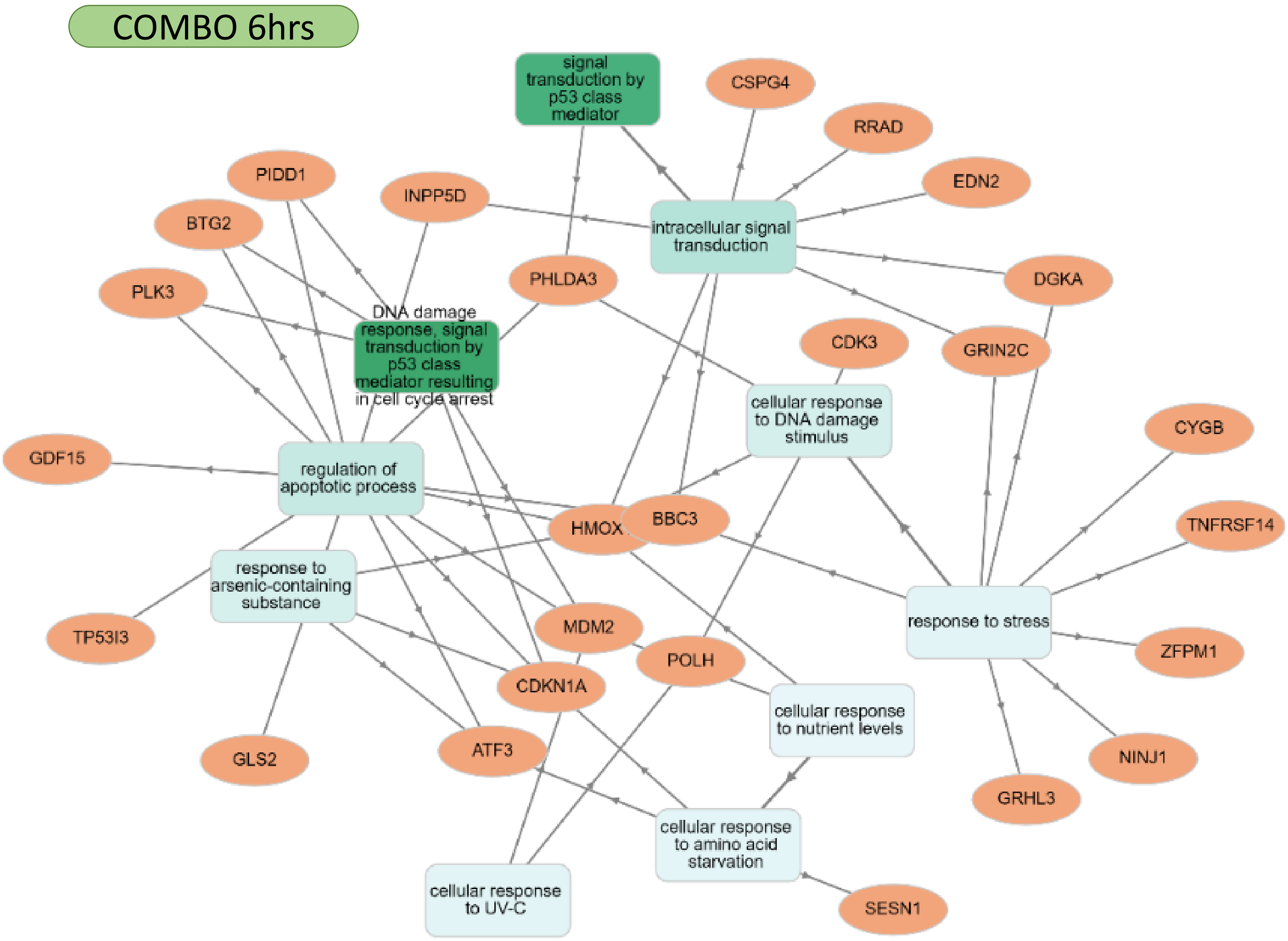

Supplementary Figure S3. RNAseq for Gene ontology (GO) enrichment analysis with Database of Immune Cell Expression (DICE). RBE were treated with HDM201 and GSK2830371 for 6 hours.

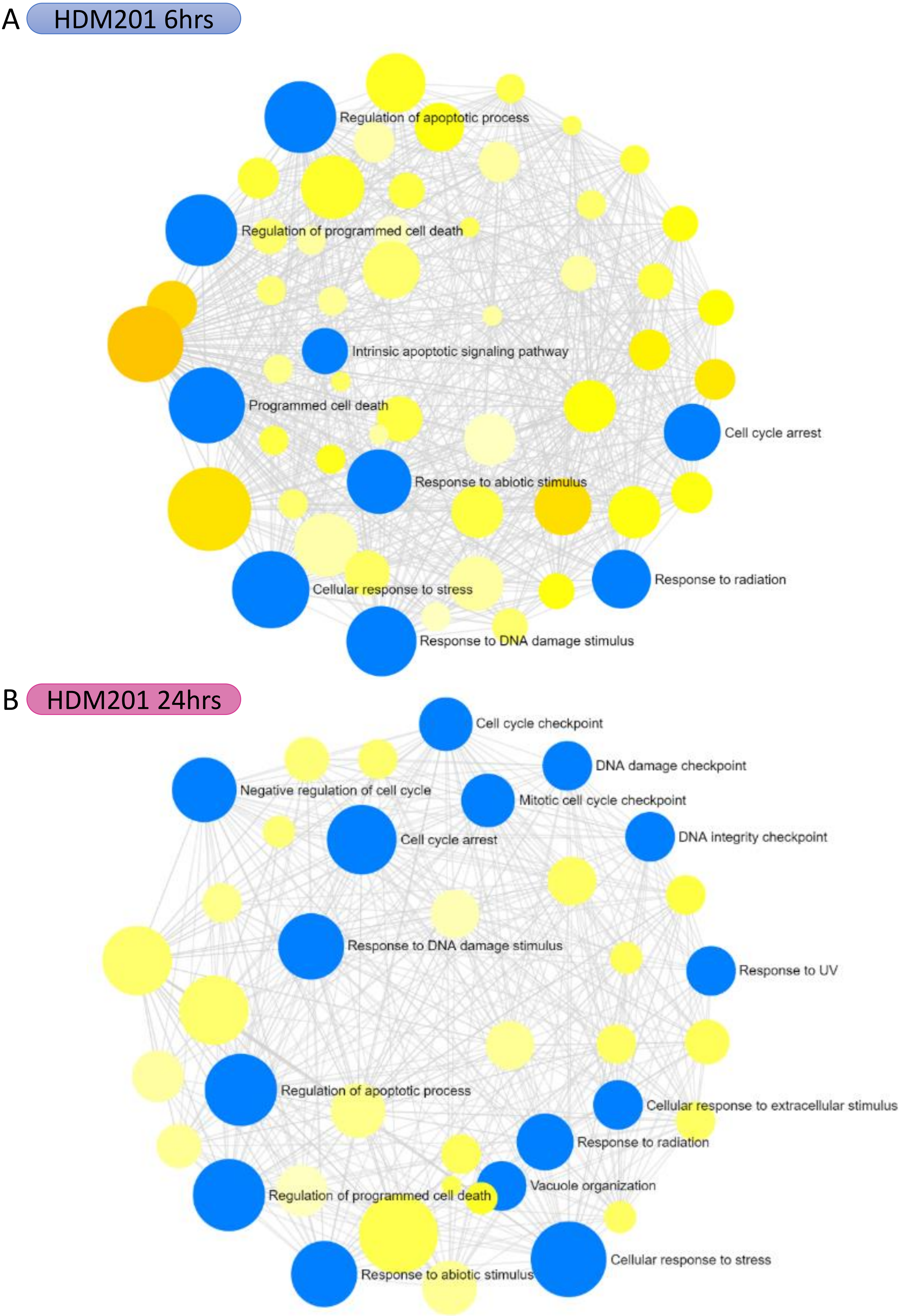

Supplementary Figure S4. RNAseq for gene ontology database in biological process. RBE were treated with HDM201 alone for 6 hours (A),24 hors (B).

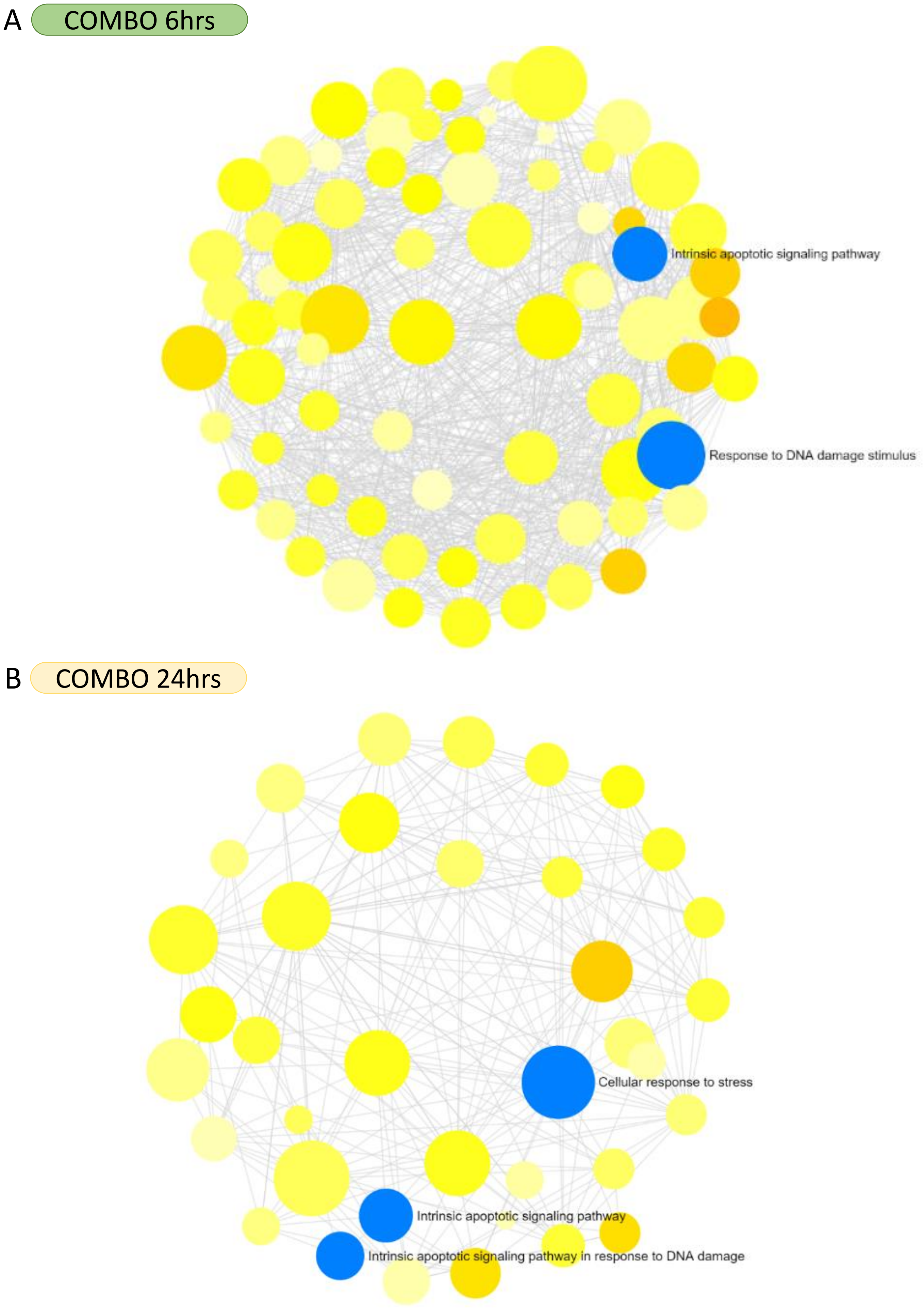

Supplementary Figure S5. RNAseq for gene ontology database in biological process. RBE were treated with HDM201 plus GSK2830371 for 6 hours (A) and 24 hours (B).

A HDM201 6hrs

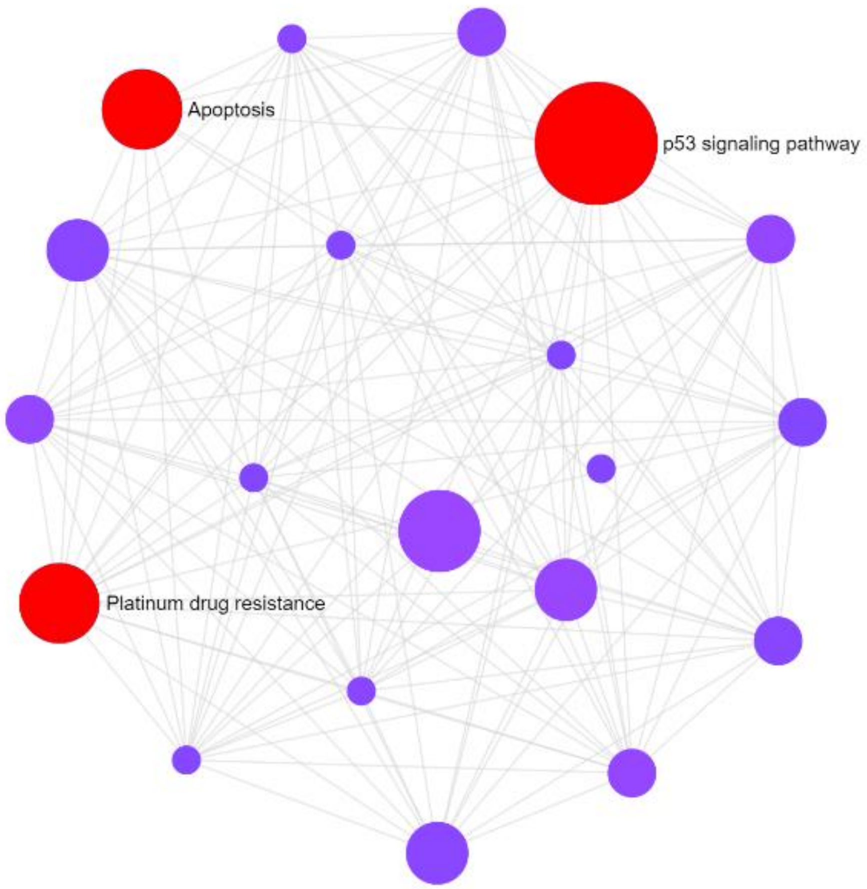

B HDM201 24hrs

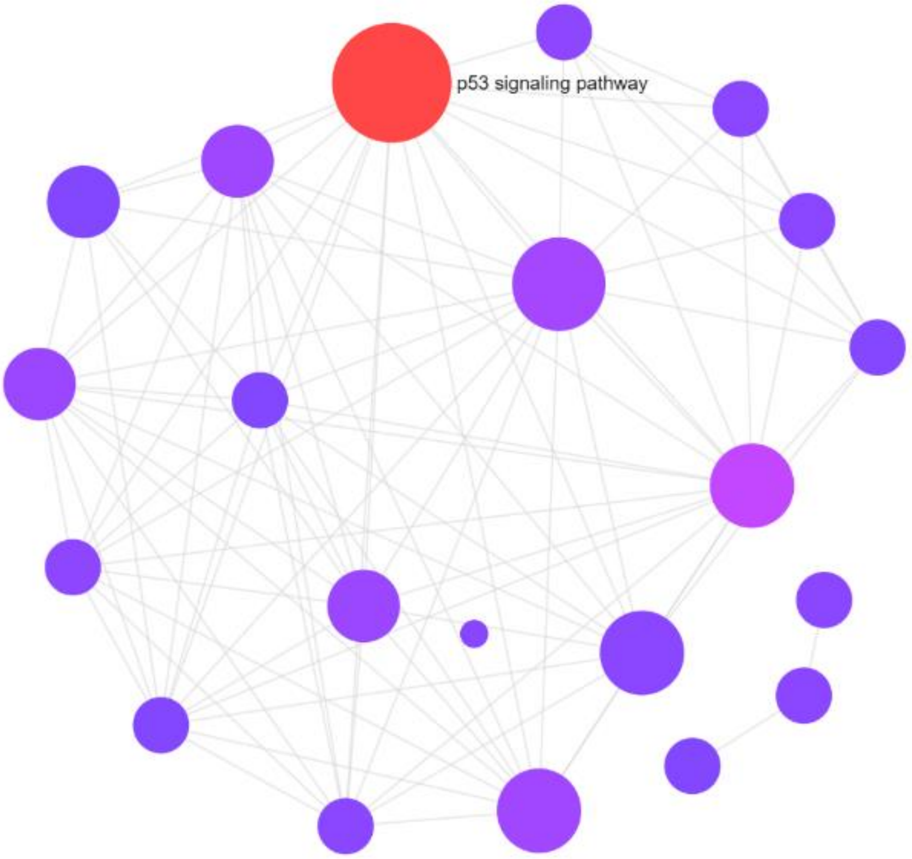

Supplementary Figure S6. RNAseq for gene ontology enrichment analysis using the KEGG pathway database. RBE were treated with HDM201 alone for 6 hours (A) and 24 hors (B).

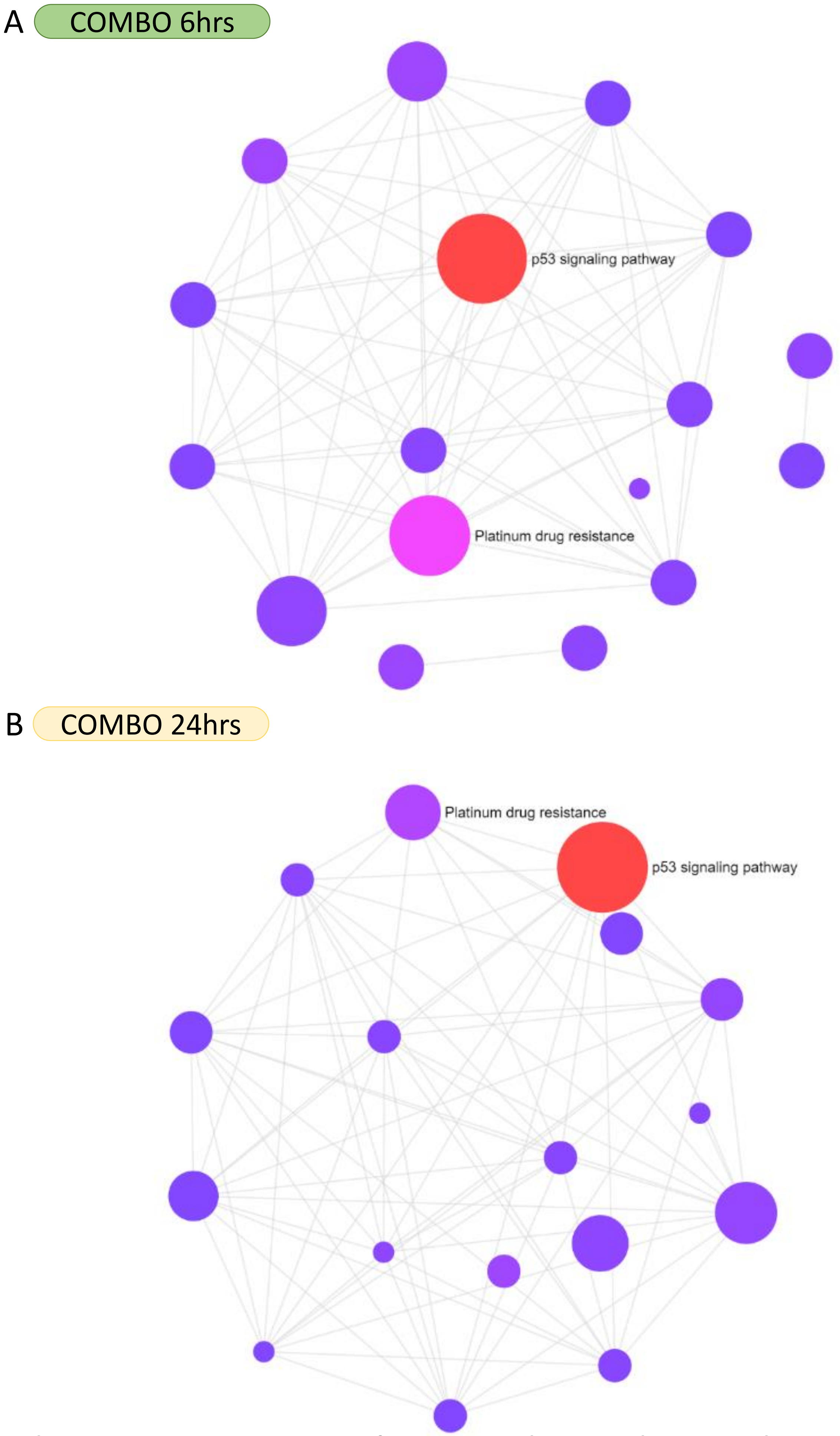

Supplementary Figure S7. RNAseq for gene ontology enrichment analysis using the KEGG pathway database. RBE were treated with HDM201 plus GSK2830371 for 6 hours (A) and 24 hours (B).

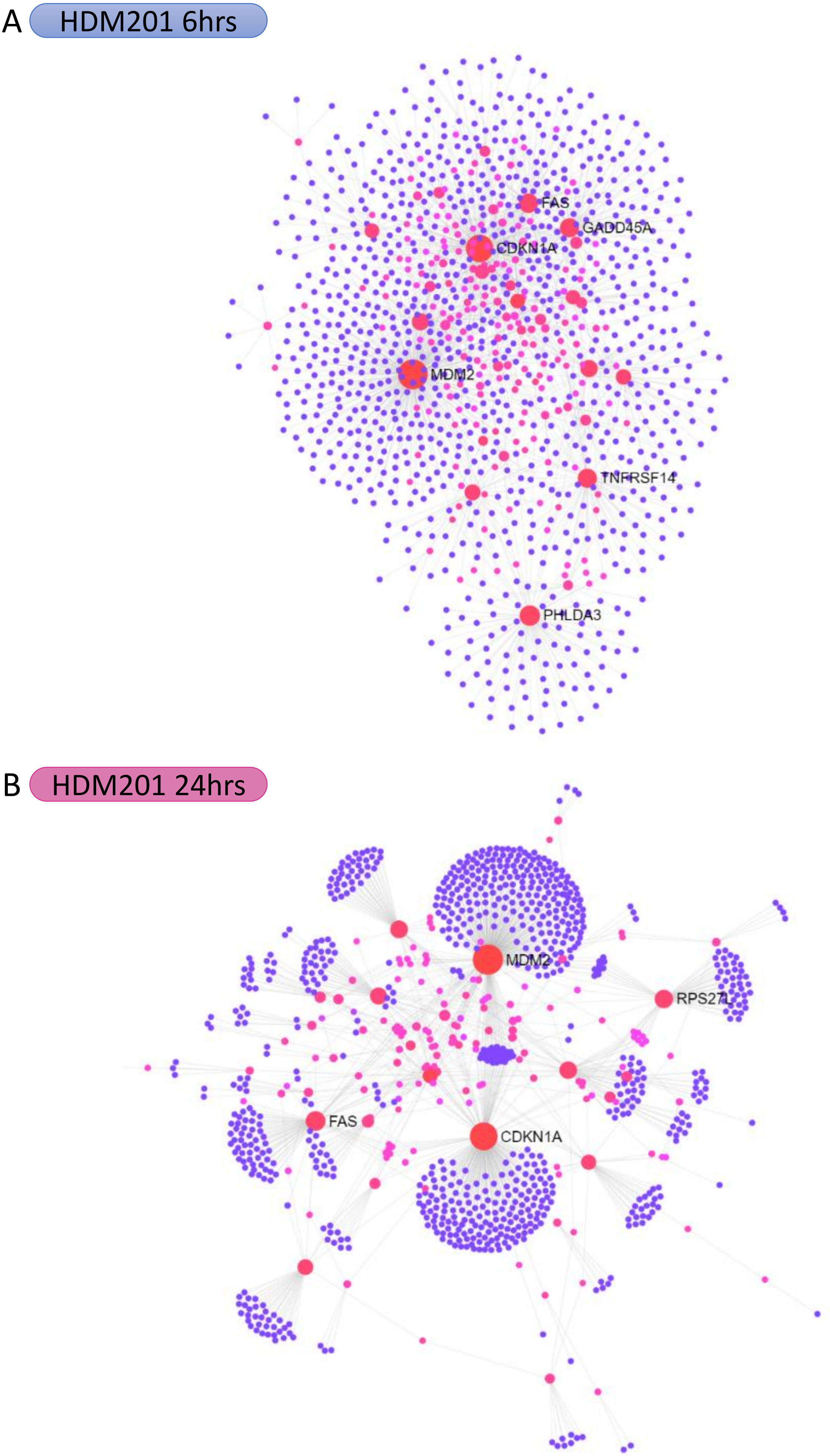

Supplementary Figure S8. RNAseq for gene ontology database for protein-protein interaction. RBE were treated with HDM201 alone for 6 hours (A) and 24 hors (B).

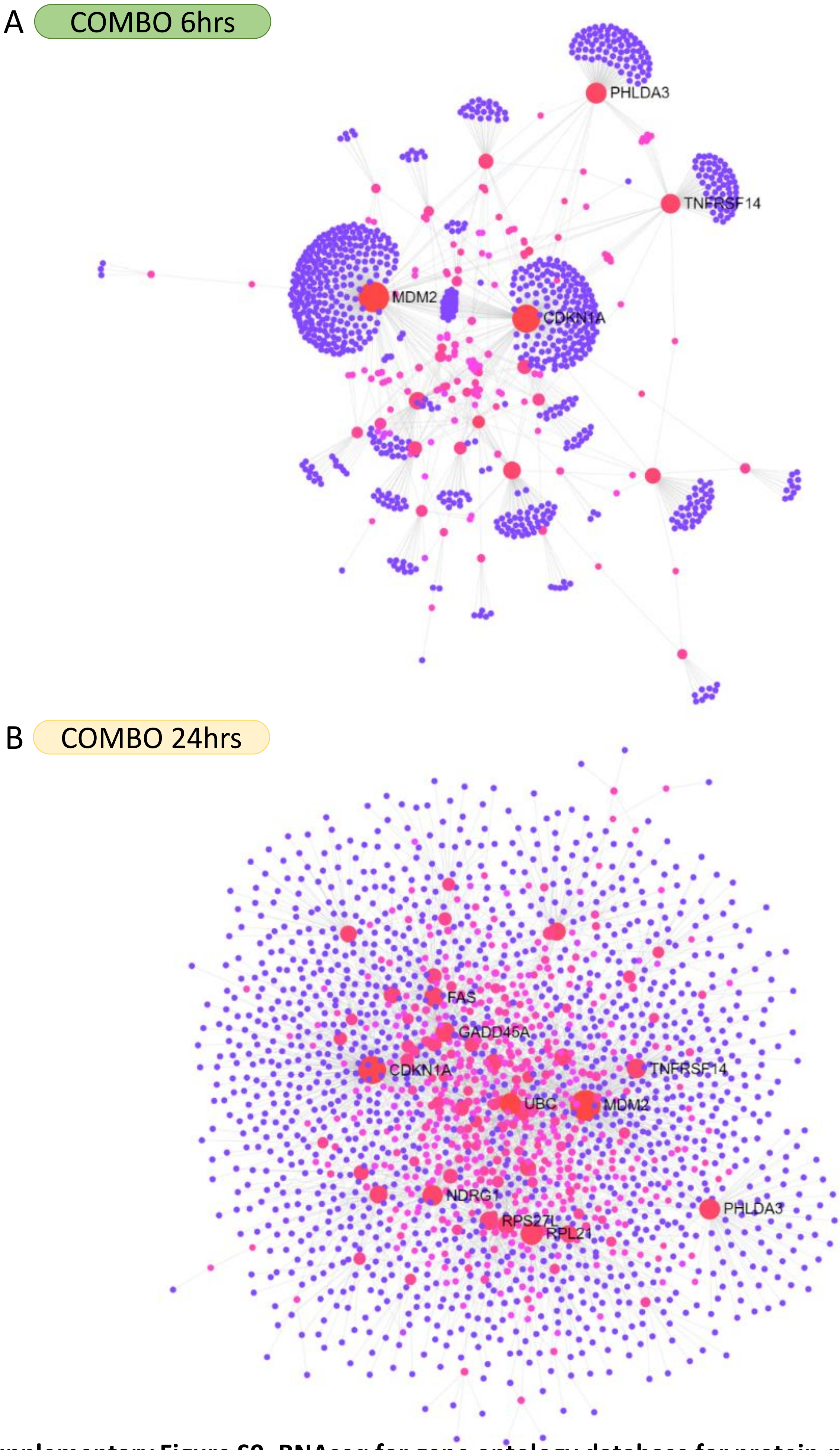

Supplementary Figure S9. RNAseq for gene ontology database for protein-protein interaction. RBE were treated with HDM201 plus GSK2830371 for 6 hours (A) and 24 hours (B).
